# Graft Suturing for Lenticule Dislocation after Descemet Stripping Automated Endothelial Keratoplasty

**Published:** 2011-04

**Authors:** Wai-Kwan Wu, Victoria W Y Wong, Stanley C C Chi

**Affiliations:** Department of Ophthalmology and Visual Sciences, Chinese University of Hong Kong and Hong Kong Eye Hospital, Hong Kong, China

**Keywords:** Corneal Transplantation, Descemet Stripping Automated Endothelial Keratoplasty

## Abstract

**Purpose:**

To report the mid-term outcomes of graft suturing in a patient with lenticule dislocation after Descemet stripping automated endothelial keratoplasty (DSAEK).

**Case Report:**

A 78-year old woman was found to have graft dislocation involving the nasal half of the cornea after uneventful DSAEK. Graft repositioning, refilling the anterior chamber with air, and placement of four full-thickness 10/0 nylon sutures over the detached area were performed two weeks after the initial surgery. The sutures were removed 6 weeks later. Serial specular microscopy and anterior segment optical coherence tomography were performed. At 18 months, there was good lenticule apposition and a clear graft.

**Conclusion:**

Anchoring sutures seem to be effective for management of graft detachment following DSAEK.

## INTRODUCTION

Descemet stripping automated endothelial keratoplasty (DSAEK) is a recently performed procedure for replacement of diseased corneal endothelium. This procedure has enabled faster visual rehabilitation and greatly reduced postoperative complications associated with conventional penetrating keratoplasty (PK), including high astigmatism and suture-related problems, and serious intraoperative complications such as suprachoroidal hemorrhage.

DSAEK, however, is associated with a new spectrum of complications unique to the procedure. Graft dislocation is one of the most common complications which has been reported in up to 82% of cases in some series.^1^ Most of these cases are managed by graft repositioning and reinjection of an air bubble into the anterior chamber. Despite these measures, up to one-third of grafts remain detached after such interventions.^2^ An alternative treatment for early graft dislocation utilizing suture fixation has been reported with good outcomes up to 6 months.^3^ We hereby report the mid-term outcomes of successful treatment of a case of lenticule detachment following DSAEK by temporary suture fixation and rebubbling.

## CASE REPORT

A 78-year old woman with angle closure glaucoma and history of laser iridotomy in 2000, and phacoemulsification with intraocular lens implantation in 2009, was found to have progressive corneal decompensation in her right eye. Visual acuity was counting fingers and pachymetry showed central corneal thickness of 772 μm. She underwent DSAEK in early 2009. Endothelial cell count of the graft was 2,849 cells/mm^2^ and the lenticule was 163 μm in thickness as determined by intraoperative pachymetry. After Descemet stripping of the recipient cornea under direct visualization in an area 8 mm in diameter, an 8 mm donor lenticule was inserted using the sheets glide technique as described by Mehta et al.^4^,^5^ Four mid-peripheral venting incisions were created to improve apposition of the graft-stromal interface. Filtered air was used to completely fill the anterior chamber for 7 minutes after graft insertion.

**Figure 1:**
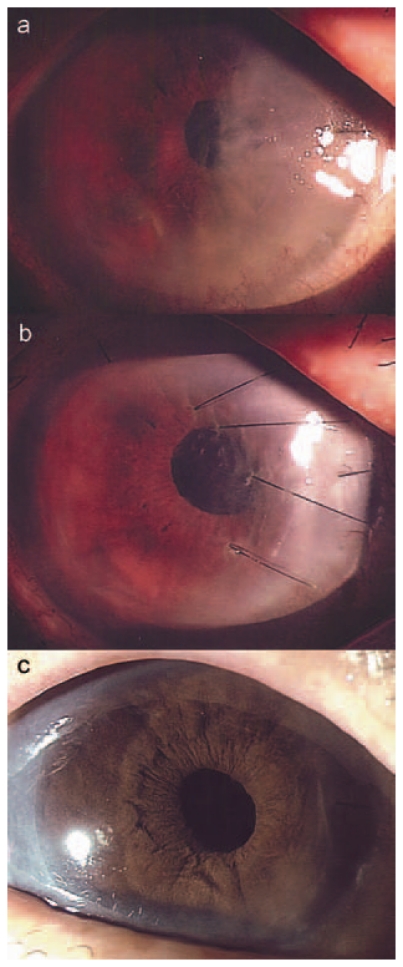
Slit lamp photographs of the right eye of the patient showing: **(a)** severe nasal corneal edema due to graft dislocation after DSAEK; **(b)** four full-thickness sutures placed to anchor the dislocated lenticule, note residual corneal folds on day 5 after reoperation; and **(c)** a clear cornea, 18 months after the operation.

**Figure 2:**
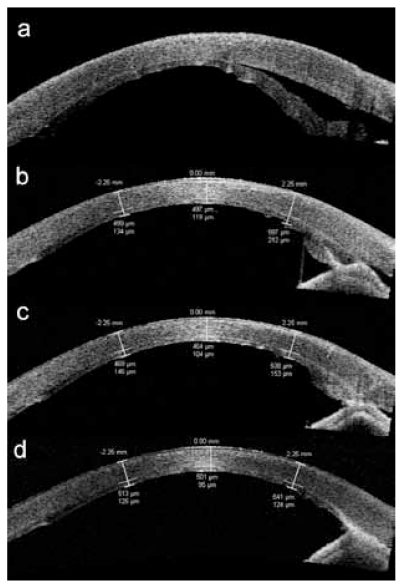
Serial anterior segment optical coherence tomographic images of the right eye of the patient (horizontal cut) showing: **(a)** nasal graft dislocation one week after DSAEK with presence of peripheral anterior synechiae; **(b)** apposition of the graft one month after suture fixation; **(c)** gradual thinning of the posterior lamella at the site of previous dislocation at 6 months; and **(d)** normal peripheral corneal thickness with a mild increase in echogenicity suggestive of scarring at 1 year, the peripheral anterior synechiae persisted despite intervention.

Postoperatively, an eye shield was used to protect the eye and the patient was advised against eye-rubbing. There was a 50% air bubble in the anterior chamber after surgery and the patient remained in face-up position for the first three days. Despite these measures, a gap between the posterior graft and the stroma was noted over the nasal cornea on the third postoperative day. Over the next week, corneal edema worsened to involve half of the cornea (Fig. 1a). Anterior segment optical coherence tomography (ASOCT; Visante, Carl Zeiss Meditec, Dublin, CA, USA) was performed and confirmed the presence of nasal graft dislocation with peripheral anterior synechiae (PAS) formation at 9 o’clock (Fig. 2a). Due to initial reluctance for reoperation by the patient, repositioning of the partially dislocated graft was deferred until 2 weeks after DSAEK. In view of the chronicity and large extent of graft detachment, it was decided that in addition to graft repositioning and anterior chamber refilling with air, full-thickness sutures be placed to stabilize the lenticule.

Reoperation was performed under local anesthesia. The dislodged graft was repositioned with a 30 G needle via the two nasal venting incisions. Air was then injected temporally 1 mm posterior to the limbus using a 30 G needle and the anterior chamber was completely filled. Four full-thickness 10/0 nylon sutures were then placed from the periphery inwards to cover the extent of graft dislocation. After the knots were buried, the anterior chamber was refilled with air for 7 minutes.

Postoperatively, the graft was apposed and by the fifth day, there were only residual corneal folds (Fig. 1b). ASOCT was performed after reoperation which demonstrated good graft apposition and gradual reduction in corneal edema; the PAS, however, persisted (Fig. 2b-d). The sutures were removed at a slit lamp after 6 weeks.

The patient was regularly monitored; 18 months postoperatively, the cornea was clear (Fig. 1c) and visual acuity remained stable at 20/60 with +1.50 −1.75 ×65º. Endothelial cell count measured by specular microscopy (Robo, Kowa, Japan) one month after the operation was 2,247 cells/mm^2^ (21% reduction). Subsequent endothelial cell counts were 1,700 cells/mm^2^, 1,204 cells/mm^2^, 1,014 cells/mm^2^, and 943 cells/mm^2^ at 3, 6, 12, and 18 months. Central corneal thickness and central posterior graft thickness measured by ASOCT were 497 μm and 119 μm at month 1, 464 μm and 104 μm at month 3, and 501 μm and 95 μm at month 12, respectively (Fig. 2b-d).

## DISCUSSION

Various complications may be encountered in DSAEK. Common ones include graft dislocation, graft rejection, primary graft failure, secondary glaucoma, endothelial cell loss, and refractive surprise.^1^ Graft dislocation is the most frequent complication and occurs at a mean rate of 14%.^1^,^6^

Causes of posterior graft dislocation include eccentric trephination, surgical trauma, delayed endothelial cell recovery, primary graft failure, presence of viscoelastic or excessive fluid in the interface, and postoperative eye rubbing.

Graft dislocations most often occur during the early period of a surgeon’s learning curve, mostly due to unnecessary manipulation during the procedure. The patient described herein, was one of the first few DSAEK procedures performed by the operating surgeon during transition from PK and was the only case of graft dislocation in the first ten cases.

Graft dislocation usually occurs in the first week after surgery, but late dislocation has been reported up to 6 weeks postoperatively.^7^ Modifications in surgical technique and postoperative management have reduced the risk of graft dislocation. Such interventions include strict advice against eye-rubbing in the early postoperative period, creation of mid-peripheral venting incisions, peripheral recipient bed scraping, leaving a large air bubble, and maintaining supine position for a longer period.^6^,^8^,^9^ Different glides have been designed to facilitate graft insertion and minimize manipulations, hence reducing endothelial cell damage and enhancing graft adherence.^10^

Lenticule dislocations are usually treated with graft repositioning and refilling the anterior chamber with air. Due to the relatively high frequency of postoperative graft dislocation, patients are usually informed preoperatively that rebubbling may be required to facilitate good apposition of the graft in order to prevent or manage graft dislocation in the early postoperative period. Suh et al^2^ reported a detachment rate of 23% in their series of 118 DSAEK cases, most of them being partial detachments. Among the 25 eyes that required further intervention, 22 patients underwent rebubbling and 3 required both repositioning and rebubbling. Despite these interventions, the graft remained detached in 32% of complicated cases and led to repeat DSAEK or PK. Of successfully apposed grafts, 24% of corneas remained edematous. In another large series of 126 eyes, Shih et al^11^ similarly reported that 22% of eyes developed graft detachment after DSAEK; 32% of these corneas failed to clear up after refloating. Occasionally, graft dislocations may spontaneously appose without intervention.^2^,^11^,^12^

Anandan and Leyland^3^ previously reported suturing as a method for managing repeated posterior graft dislocation. In their series of three patients, four to six fixation sutures were placed in addition to repositioning and refilling the anterior chamber with air. The grafts were successfully apposed and there was significant visual improvement in all cases. We believe that the additional placement of sutures may facilitate attachment in cases involving a large area of detachment and chronic cases, as in this patient. This surgical alternative should also be considered in patients with redetachments.

This case report provides additional information by documenting endothelial cell loss beyond the first year. The initial reduction in endothelial cell count was 21% one month after intervention. Subsequently there was a gradual reduction in endothelial cell count over the next 17 months, with a final cell count of 943 cells/mm^2^ at month 18, resulting in a cumulative cell loss of 67%. Endothelial cell loss at one year has been reported to range from 24% to 61%.^1^ It is postulated that excessive cell loss may be the result of prolonged detachment, therefore earlier intervention may be beneficial.

The patient described herein was an elderly woman with concomitant narrow angle glaucoma and a relatively small eye with a shallow anterior chamber. Even though a small 8 mm graft was inserted, the peripheral dislocation and edema in the posterior lenticule caused PAS formation at the site of contact.

In summary, we report the mid-term outcomes of intervention for a case of graft dislocation two weeks after DSAEK which was successfully apposed by placement of fixation sutures. The procedure is simple and seems to be suitable for eyes in which reapposition is difficult, or with chronic graft dislocations. This technique might improve reattachment and reduce primary graft failure rates, thus decreasing the need for regrafting. A study comparing rebubbling alone and rebubbling combined with suture placement would be useful.
